# H-GRASP: the feasibility of an upper limb home exercise program monitored by phone for individuals post stroke

**DOI:** 10.3109/09638288.2016.1162853

**Published:** 2016-03-26

**Authors:** Lisa A. Simpson, Janice J. Eng, May Chan

**Affiliations:** ^a^ Graduate Program in Rehabilitation Sciences, University of British ColumbiaVancouverCanada; ^b^ Rehabilitation Research Program, GF Strong Rehab Centre, Vancouver Coastal Health Research InstituteVancouverCanada; ^c^ Department of Physical Therapy, University of British ColumbiaVancouverCanada; ^d^ Occupational Therapy Department, Abbotsford Regional HospitalAbbotsfordCanada

**Keywords:** Rehabilitation, stroke, telemedicine, upper limb

## Abstract

**Purpose**: To investigate the feasibility of a phone-monitored home exercise program for the upper limb following stroke. **Methods**: A pre-post double baseline repeated measures design was used. Participants completed an 8-week home exercise program that included behavioural strategies to promote greater use of the affected upper limb. Participants were monitored weekly by therapists over the phone. The following feasibility outcomes were collected: Process (e.g. recruitment rate); Resources (e.g. exercise adherence rate); Management (e.g. therapist monitoring) and Scientific (e.g. safety, effect sizes). Clinical outcomes included: The Chedoke Arm and Hand Inventory, Motor Activity Log, grip strength and the Canadian Occupational Performance Measure. **Results**: Eight individuals with stroke were recruited and six participants completed the exercise program. All but one of the six participants met the exercise target of 60 minutes/day, 6 days/week. Participants were stable across the baseline period. The following post-treatment effect sizes were observed: CAHAI (0.944, *p =* 0.046); MALQ (0.789, *p =* 0.03) grip strength (0.947, *p =* 0.046); COPM (0.789, *p =* 0.03). Improvements were maintained at three and six month follow ups. **Conclusions**: Community dwelling individuals with stroke may benefit from a phone-monitored upper limb home exercise program that includes behavioural strategies that promote transfer of exercise gains into daily upper limb use.

**Implications for Rehabilitation**
- A repetitive, task-oriented home exercise program that utilizes telephone supervision may be an effective method for the treatment of the upper limb following stroke
- This program is best suited for individuals with mild to moderate level impairment and experience a sufficient level of challenge from the exercises
- An exercise program that includes behavioural strategies may promote transfer of exercise gains into greater use of the affected upper limb during daily activities

## Introduction

Following a stroke, up to three quarters of survivors will experience upper limb impairments that may impact their ability to participate in life activities.[1] At six months post stroke, less than half of these individuals will regain independent functioning of their upper extremity.[2] The discrepancy between what a patient can do and actual use of the paretic limb in the patients’ own environment, referred to as learned non-use, is also a major concern.[3] Despite gains made during rehabilitation, many individuals do not go on to use their paretic upper limbs.[4,5] Reduced arm use has been associated with decreased strength, bone density and reduced quality of life.[6,7] Therefore, maximizing lasting rehabilitation gains is important for the patients’ long-term health and for a sustainable healthcare system.

Interventions with the best evidence for promoting upper limb recovery after stroke involve intensive, repetitive and task-oriented practice.[8] The Graded Repetitive Arm Supplementary Program (GRASP) is a recently developed self-administered intervention for the paretic upper extremity that is based on these evidence-based principles. A 2009 study found GRASP to be effective for increasing upper limb function and use during inpatient rehabilitation and GRASP has been widely implemented.[9–11] Participants who received the GRASP protocol were asked to perform 60 minutes of exercise per day outside of structured therapy time and were monitored weekly by a therapist. The self-administered design of GRASP allows it to be cost-effective with inexpensive equipment and minimal increases in direct therapist time. We propose that the GRASP design may allow it to be adaptable for use in the home with community dwelling individuals with stroke.

While the original GRASP inpatient trial utilized face-to-face sessions by a therapist for the purpose of progressing the exercises and to monitor adherence, a home program could potentially utilize phone monitoring to check whether exercises are being completed and whether the patient is incorporating their stroke-affected arm into their daily activities. A program initiated in the home has advantages; one could more easily transfer the gains made from the repetitive exercises to using one’s affected arm and hand during daily activities (and thus, circumvent learned non-use). Also, as hospital stays continue to reduce, patients are being discharged home earlier where they may not have access to intensive therapies. Current literature calls for more resource efficient interventions to continue therapy in the home and/or reach rural patients who may not have access to outpatient care.[8]

To date, there are few studies that examine home based exercise programs for the stroke affected upper limb. A recent Cochrane meta-analysis found there was insufficient evidence to determine whether home-based exercise programs had an effect on upper limb recovery.[12] A 2014 study however utilized a three-month home exercise program and a tele-rehabilitation component and found increased upper limb strength and function following the intervention.[13] It is unclear if the participants in this study translated their strength and functional gains into greater use of their upper limbs however as the study did not include a measure of upper limb use.

In addition, few existing treatments incorporate behavioural change strategies to address the propensity of learned non-use. Behavioural change interventions that utilize self-management approaches, which involve the active participation of clients, have been effective for increasing healthy behaviours such as physical activity.[14] Moreover, behavioural strategies that incorporate self-regulation techniques such as goal setting, self-monitoring of behaviour and feedback on performance are particularly effective.[14] A recent systematic review examining the use of self-management programs for people with stroke found preliminary support for self management programs specific to the stroke population. However, the review noted that the exact timing, content and delivery mode still needs to be determined.[15] One example of an intervention that utilizes behavioural change strategies to promote an increase in upper limb use is Constraint-Induced Movement Therapy (CIMT).[16] Although CIMT has strong evidence to support its efficacy, it is both resource and time intensive. Investigations into more cost-efficient interventions that incorporate behavioural strategies are therefore warranted.

We developed a novel program called the H-GRASP (Home-GRASP) with the following attributes: (1) graded self-administered exercises adapted from GRASP; (2) use of behavioural strategies to promote incorporation of the paretic limb into daily activities; (3) support and monitoring provided by phone. The objective of this study was to investigate the feasibility of the H-GRASP among individuals living in the community following a stroke.

## Methods

**Figure 1.**
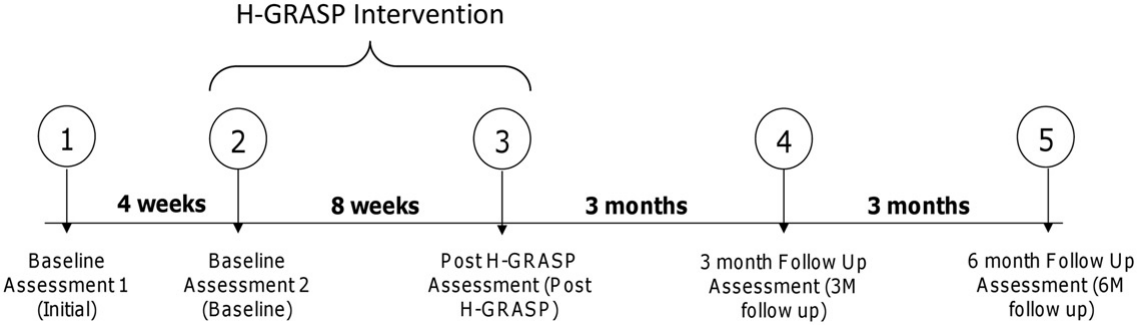
Study design.

This study used a repeated measures design; a double baseline measurement was followed by the eight-week intervention and three follow-up measurements. Participants were assessed at the following time periods: upon recruitment (initial), four weeks post initial assessment (baseline); post H-GRASP training; (post H-GRASP); three months post training (3 M follow up) and six months post training (6 M follow up) (Figure 1). The study was approved by the relevant university ethics board.

### Participants

Potential participants were recruited from outpatient stroke rehabilitation programs at two hospitals. The inclusion criteria was: (1) >19 years old; (2) diagnosis of first-ever stroke, confirmed by magnetic resonance imaging (MRI) or computed tomography (CT) (3) >2 months and <12 months post stroke; (4) 10° active wrist and finger extension (4) difficulty using the affected upper limb (<5 on the Motor Activity Log); (5) completion of formal rehabilitation programs; (6) living in the community (7) able to understand and follow instructions. Exclusion criteria included: (1) neurological condition other than stroke; (2) medically unstable; (3) musculoskeletal condition that affected use of either UE (e.g. fracture or arthritis); (4) previous experience with the GRASP protocol, (5) no identified upper limb therapy needs as identified by the treating outpatient occupational or physical therapist. All participants were screened for eligibility during a telephone session prior to participating. Individuals with severe communication impairment that limited their ability to communicate over the phone were not excluded from the study if a caregiver was able to participate.

### Intervention

The original GRASP (http://neurorehab.med.ubc.ca/grasp/) consisted of three sets of exercises (Level 1, 2 and 3) that coincided with severe, moderate and mild impairment levels according to the Fugl-Meyer Impairment Scale. H-GRASP consists of a single set of graded exercises formed by collapsing Levels 2 and 3 of the original GRASP. Research suggests that patients who possess severe upper limb impairments at two months post stroke have decreased potential for exercise-mediated improvements.[17] Therefore Level 1 was excluded from H-GRASP and the minimal level required was 10° of active wrist and finger extension. H-GRASP exercises focus on range of motion, strengthening, weight bearing, and fine and gross motor skills. Repetitive task-oriented tasks were included for practice of partial or whole skill sets required to perform functional activities (e.g. buttoning, pouring and lifting). Participants were taught the exercises during one training session at the local hospital by the occupational therapists trained in the H-GRASP protocol. At this session participants received a binder containing written and pictorial instructions for each exercise as well as a kit which contained inexpensive items (e.g. ball, paper clips, clothes pegs) to complete the exercises.

For most exercises, there were two recommended progressions of repetitions (e.g. 2 sets of 5, 3 sets of 10). If the patient could complete all the repetitions without fatigue or error, then it was recommended that the task itself be altered (e.g. reduce size of object to manipulate) to create a participant’s “just right challenge”. Thus the therapists tried to ensure that each exercise was challenging enough to elicit a training effect but not too difficult that the participant would become frustrated or use gross compensations (i.e. excessive shoulder elevation, trunk rotation). However, there was also responsibility given to the patient to increase the difficulty of the task. In multiple sections of the exercise binders, there was emphasis on the need to make the task challenging. For example, the participant binder had a full page in the middle of the book, which would have been observed in each session that stated the following:

These hand exercises need to be challenging to improve your brain’s function. If you completed a hand exercise set without making any mistakes such as dropping the object, you need to make it more difficult. Here are some ideas to increase the challenge: use smaller lego blocks, use toothpicks for pick-up sticks, use dimes for the flip-over exercise, use smaller paper clips for paper clip exercise, use small blocks for block towers, try to do the exercises faster. The exercises should be so difficult that you drop or mishandle an object one out of every five repetitions.

To support the patient in progressing the exercises, the GRASP kit consisted of at least two sets of items for most exercises (e.g. small and large set of lego blocks).

Participants were asked to complete the exercises for 60 minutes/day, 6 days a week for 8 weeks. This is the duration and frequency of exercise identical to the GRASP protocol used for individuals undergoing inpatient stroke rehabilitation. The original GRASP trial observed improvements in participants’ arm function after a four-week program. An eight-week program length was selected for H-GRASP based on the expectation that changes in upper limb function would take longer in a more chronic population. Also, upper limb exercise trials for community dwelling individuals with stroke observed upper limb function improvements with programs lasting 6–19 weeks.[18–20] Participants were asked to keep a daily log to track the number of repetitions and sets of each exercise and the number of days and total amount of time per day spent completing H-GRASP exercises. The therapists called the participants weekly to monitor adherence and progress the program as needed. To progress the exercises, the therapists asked the participants to describe how easy it was to perform each exercise as prescribed. Exercises were progressed (i.e. increasing repetitions, sets and/or amount of weight) if the participant could perform the exercises straight through without a break or without errors (i.e. dropping an object).

A number of behavioural strategies were built into the protocol to ensure exercise adherence and to promote integration of the paretic upper limb into daily activities. The behavioural strategies built into H-GRASP are outlined in Table 1. In summary, H-GRASP included the use of behavioural contracts, confidence forms, social support, goal setting and feedback on amount of upper limb use.[14]

**Table 1.** H-GRASP behavioural-change techniques.

Protocol Features	Explanation	Behavioural Change Technique
Weekly monitoring	Therapists made weekly phone calls to participants to inquire about adherence to program, ascertain level of challenge and progress the program as needed	Prompt self-monitoring; prompt feedback on performance
Identify weekly task goals	Therapists and participants worked collaboratively to identify weekly task goals for increasing upper limb use during daily activities	Prompt goal setting
Monitoring completion of task goals	Therapists reviewed participants' experiences in completing weekly task goals	Prompt review of behavioural goals
Prompting % arm use each week	Therapists prompted participants to report the percent of time they were using their affected upper limb during daily activities	Prompt feedback on performance
Identify barriers and assist with problem solving	Therapists prompted participants to identify barriers to completing exercise targets and task goals. Assisted participants to problem solve solutions for overcoming barriers (ie. split sessions up into manageable time blocks, ensure a mix of challenging and less challenging task goals)	Prompt barrier identification
Encourage family/caregiver involvement	Participants were encouraged to invite caregivers to training session and be involved with exercise program and upper limb task goals	Prompt social support
Behavioural contract	At H-GRASP training session, participants were asked to sign a behavioural contract in which they agreed to adhere to exercise targets and attempt performance of task goals	Behavioural contract
Identify level of confidence	Participants were asked to identify their level of confidence to adhere to exercise targets. If confidence <8/10, therapist and participant discussed facilitators and barriers to adherence and problem solved strategies for overcoming barriers.	Prompt intention formation

Behavioural change techniques were identified and attached to each protocol feature using behavioural technique codes and definitions from Michie et al.[14,39]

### Outcomes

Demographic and stroke characteristics were recorded upon recruitment. Feasibility outcomes were organized into the following four feasibility classifications: process, resources, management and scientific.[21] Table 2 outlines the feasibility outcomes and thresholds for success captured within the four categories.

**Table 2.** Feasibility outcomes.

Feasibility indicator	Outcome	Criteria for success	Result	Success (Y/N)
**Process**				
Recruitment Rate	% of individuals approached who were eligible and agreed to participate	20%	8/30 (26%)	Y
Retention Rate	% participants who completed 8 week H-GRASP intervention	>80% participants complete program	6/8 (75%)	N
Perceived benefit	Exit questionnaire from participants who completed the program	>80% responses at least “4/5”	6/6 (100%)	Y
**Resources**				
Exercise Adherence Rates	% participants who achieved 360 minutes of average weekly exercise	>80% participants	6/8 (75%)	N
Weekly Task Goal Adherence Rates	% participants who attempted ≥ 1 task goal each week	>80% participants	6/8 (75%)	N
**Management**				
Therapist monitoring of participant goal tasks	Prompt participant to report status of task goals (i.e. attempted or not);	80% of sessions	49/52 (94%)	Y
	Prompt participant to generate new task goals	80% of sessions	49/52 (94%)	Y
Promoted feedback on performance	Prompted for weekly % use value	80% of sessions	48/52 (92%)	Y
**Scientific**				
Safety	# of participants with increased pain while performing exercises (as measured by visual analogue scale)	No participants with increased pain while performing exercises at week 8	0/6	Y
Treatment Efficacy	Effect size of the primary and secondary outcomes	n/a		n/a
	CAHAI	n/a	0.944	n/a
	MAL	n/a	0.789	n/a
	Grip strength	n/a	0.947	n/a
	COPM	n/a	0.789	n/a
	% who get over the MCID	n/a		n/a
	CAHAI	n/a	33%	n/a
	MAL	n/a	50%	n/a
	Grip strength	n/a	33%	n/a
	COPM	n/a	50%	n/a

CAHAI: Chedoke Arm and Hand Inventory; MAL: Motor Activity Log; COPM: Canadian Occupational Performance Measure; MCID: Minimally Clinical Important Difference. Note: the MCID values are: 7.1 points for CAHAI;[18] 1.0 point for MAL;[26] 5.0 kilos for grip strength [26] and 1.7 points for COPM.[27]

### Process, resources and management feasibility

Recruitment rate, retention rate and perceived benefit were used to assess process feasibility. Perceived benefit of the H-GRASP program was measured using an exit questionnaire. Participants who completed the H-GRASP intervention were asked to rate the program’s ease of use and benefit on a five point scale with lower numbers indicating lower perceived ease of use and benefit. Participants were also asked if they would recommend the program to others (yes/no). The total score ranges from 2–11. Exercise adherence and weekly task goal adherence were used to assess resource feasibility. Weekly task goals involved identifying activities participants could attempt to facilitate greater upper limb use during daily activities. Management feasibility was assessed by considering how often therapists monitored the participants’ weekly task goals for increasing use and how often they prompted participants to report their amount of use.

### Scientific feasibility

Safety and treatment efficacy were used to assess scientific feasibility. Arm and shoulder pain was assessed throughout the intervention using an interval scale ranging from 0 (no pain) to 10 (extreme pain) and was used to assess safety of H-GRASP. Treatment efficacy was assessed using one primary and three secondary clinical outcome measures. All clinical assessments were performed by trained occupational therapists at the local hospital. All but one clinical measure was conducted at all time points. All the clinical outcome measures have established reliability and validity for individuals with stroke.[22–26]

#### Primary outcome

The primary outcome was upper limb function measured by the Chedoke Arm and Hand Inventory-9 (CAHAI).[19,22] Participants were asked to complete nine daily activities that involve the use of both limbs (e.g. opening a jar, pouring a glass of water). Each task was scored on a 1–7 scale with higher scores indicating the task was performed using a greater percent contribution of the affected hand.

#### Secondary outcomes

The Motor Activity Log-14 (MAL) was used to assess affected upper limb use.[23] The MAL is a self-report measure that asks participants to rate how much (amount scale) and how well (quality of movement scale) they use their affected upper limb during 14 daily living tasks (e.g. buttoning a shirt, carrying an object, brushing teeth). Each task is rated on a scale of 1–5 with higher scores indicating greater use. Scores are averaged across the activities to obtain a final score. Only the MAL quality of movement scale was used due to its high reliability and strong correlation with the amount of use scale.[23]

The Jamar dynamometer was used to measure isometric strength of the affected hand.[21] All participants were placed in the standardized position for the test. Participants were seated in a chair with back support, shoulder at 0° flexion and rotation, elbow flexed to 90° with 0° supination/pronation and wrist in 0–30° extension. Participants were asked to squeeze the dynamometer as hard as they can for 3 s. The average of three trials was recorded.

The Canadian Occupational Performance Measure (COPM) was used to assess occupational performance.[25,26] The COPM is a self-report measured designed to capture change in self-perceived performance of activities of daily living over time. Participants were asked to rate their current level of performance on the five most important activities (relevant to the upper limbs) that cause them difficulty. Each activity is rated on a scale from 1–10 with higher scores indicating greater self-perceived performance. The final score consists of the average across the activities. The COPM was administered at baseline and post GRASP time points because priority activities may change with longer follow up periods.[27]

### Data analysis

Descriptive analysis was used to summarize baseline demographic and clinical information of the subjects. A Skillings–Mack’s test was used to examine differences in clinical scores across the five time points. The Skillings–Mack test is a non-parametric generalization of the Friedman test and is appropriate for repeated measures analyses with small sample sizes and missing data.[25,28] *Post hoc* Wilcoxon tests were conducted when significant main effects were found. The following four *post hoc* comparisons were conducted: (1) initial versus baseline; (2) baseline versus post-treatment; (3) post-treatment versus 3-month follow up and (4) post-treatment versus 6-month follow up. A Wilcoxon test was used to examine the difference between baseline and post H-GRASP COPM scores. Nonparametric Cohen’s d effect sizes based on output from the Wilcoxon tests were calculated to estimate the magnitude of the treatment effect. In addition, the number of participants who surpassed the cited minimal clinically important difference or minimally detectable change values (MCID) were calculated for the primary and secondary outcomes. The following equations were used to calculate effect sizes [29]: 2r 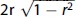 where 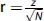 and *z* is the *z* statistic obtained from the Wilcoxon tests. All analyses were completed using Stata 14.0 (College Station, TX).

## Results

### Process feasibility

**Figure 2:**
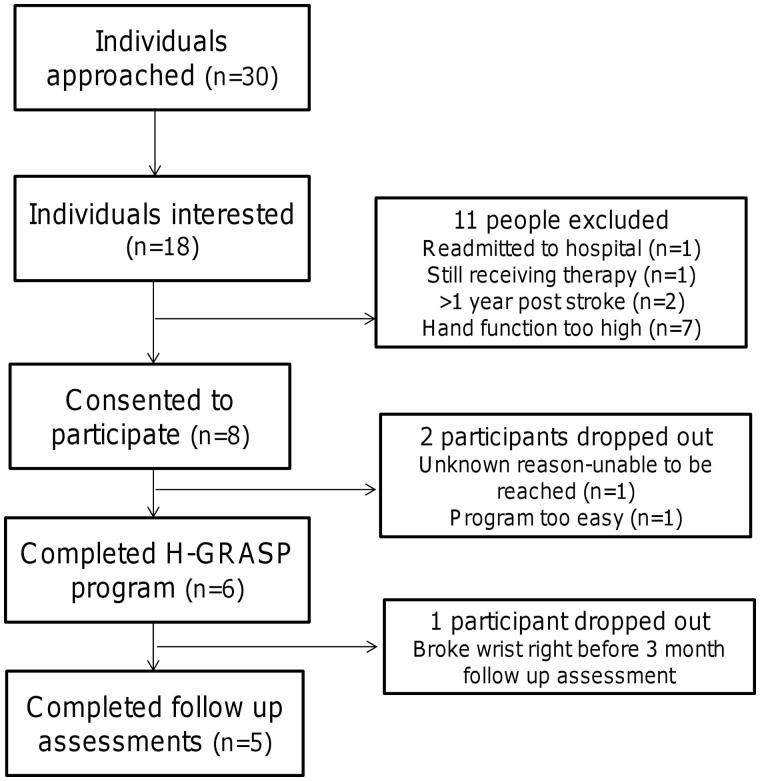
Participant flow diagram.

A total of 30 individuals were approached to participate in the study. Eight subjects who agreed to participate in the study met the inclusion criteria and provided written consent. The total recruitment rate was 26% (8/30) (Table 2). Two participants dropped out of the study after two weeks of the H-GRASP intervention. One participant dropped out for unknown reasons (participant and family contact would not return follow up phone-calls) and the other participant found the program insufficiently challenging. Thus a total of six out of eight participants (75%) completed the H-GRASP program (Table 2). Table 2 presents the feasibility results for all eight individuals. One participant fractured his wrist just before the three month follow up assessment and was unable to complete the three month and six month follow up assessments. The participant flow diagram is displayed in Figure 2. The demographic and baseline clinical characteristics of the eight participants included in the study are in Table 3.

**Table 3.** Participant characteristics.

Characteristic *N* = 8	
Age, mean (SD; range)	66.4 (7.8; 53–76)
Females, *N* (%)	4 (50%)
Days Post Stroke, mean (SD; range)	273.0 (64.2; 168–347)
Right Side Affected, *N* (%)	7 (87.5%)
Dominant Side Affected, *N* (%)	7 (87.5%)
Caregiver assistance with H-GRASP, *N* (%)	2 (25%)
Grip Strength, *N* (SD; range)	10.5 (6.9; 3.8–24.3)
CAHAI, *N* (SD; range)	33.8 (12.8; 17–55)
MALQ, *N* (SD; range)	1.6 (0.7; 0.4–2.6)

CAHAI: Chedoke Arm and Hand Activity Inventory (0–63); MALQ: Motor Activity Log Quality of Movement Scale (0–5).

All participants who completed the program believed the program was of benefit to them and all would recommend the program to others. The majority of individuals (5/6) felt the exercise booklet was easy to follow. Overall, the average score on the exit questionnaire was 10.2 with all subjects scoring at least nine out of a maximum 11 (Table 2). The two individuals who dropped out of the study before completing the program did not fill out an exit questionnaire.

### Resource feasibility

Of the participants who completed the H-GRASP, all but one participant met the exercise target of 360 minutes/week (i.e. 60 min × days). Average weekly minutes ranged from 295 to 592 (Table 4). One of the two participants who dropped out of the intervention met the exercise target while the other participant who dropped out did not. Thus the exercise adherence rate for the individuals who completed the program was 83% (5/6) and the total adherence rate was 75% (6/8) (Table 2).

**Table 4:** Clinical outcomes and exercise minutes.

	Clinical Measures (*N* = 6)*					
Average weekly minutes (*N* = 8)	Mean (SD; range)	Initial	Baseline	Post H-GRASP	3M Follow up	6M Follow up
472.2 (107.1; 295–592)	Grip Strength†‡ (kg)	7.3 (2.9; 3.8–11.1)	8.0 (2.8; 5.2–11.6)	10.6 (3.6; 5.0–14.0)	12.0 (3.8; 6.2–16.7)	13.4 (3.7; 7.7–17.3)
	CAHAI†	28.8 (9.8; 17–42)	33.5 (13.7; 18–49)	41.3 (18.4; 17–60)	42.7 (18.1; 16–62)	44.9 (18.3; 19–63)
	MALQ†	1.4 (0.6; 0.4–2.2)	1.8 (0.7; 1.0–2.8)	2.7 (1.1; 1.1–4.2)	2.6 (0.9; 1.0–3.8)	2.5 (1.0; 0.7–3.5)
	COPM§	NA	3.2 (1.8; 1.8–6.5)	5.1 (2.2; 2.4–8.2)	NA	NA

CAHAI: Chedoke Arm and Hand Inventory (0–63); MALQ: Motor Activity Log, Quality of Movement Scale (0–5); COPM: Canadian Occupational Performance Measure (1–10).

*Values are shown for individuals who completed the 8 week H-GRASP. One individual was excluded from the three month and six month assessments as he broke his wrist prior to the three month assessment date.

†*Post-hoc* Wilcoxon Signed Rank test revealed significant difference between baseline and Post H-GRASP scores (Grip Strength, *p =* 0.046; CAHAI, *p =* 0.046; MALQ, *p =* 0.03).

‡*Post-hoc* Wilcoxon Signed Rank test revealed significant difference between Post H-GRASP and 6M Follow up scores, *p =* 0.043.

§Wilcoxon Signed Rank test revealed significant difference between baseline and Post H-GRASP scores, *p =* 0.03.

With guidance from the therapists, participants identified numerous activities they could perform for greater incorporation of the affected upper limbs into daily life. Therapist guidance was influenced by a patient’s functional level and his/her upper limb goals identified on the COPM. The goal tasks ranged from reach and grasp tasks with minimal hand manipulation (e.g. wiping up a spill, flipping a light switch, opening a lever door) to tasks with greater hand manipulation (e.g. shuffling cards, using a key, writing). Overall, six out of eight (75%) participants practiced at least one task goal each week (Table 2). Five out of six participants (83%) who finished the eight-week intervention practiced at least one task goal every week.

### Management feasibility

The weekly follow up phone calls ranged in duration from 10–30 minutes. Overall, therapists prompted the participants to report whether they attempted the previous week’s task goals and prompted them to generate new goals 94% of the time (49/52 sessions). Therapists also prompted participants to report their percentage use values 92% of the time (48/52 sessions) (Table 2). Starting percentage use values of the affected upper limb reported by participants ranged from 1 to 50% and reported percentage use values at the end of the H-GRASP program ranged from 15–95%.

### Scientific feasibility

Two participants experienced pain during the H-GRASP intervention period with pain scores ranging from three to eight out of a maximum 10. Therapists assisted these participants to problem solve in order to complete the exercises without pain (i.e. remove use of weights, use table as support etc). By the end of the eight-week program, both participants were able to meet the exercise targets and did not complain of pain while completing the program.

There was a significant main effect for upper limb function captured by the CAHAI across the five time points (Skillings–Mack statistic (SM) = 14.47, *p =* 0.006). *Post hoc* Wilcoxon signed rank testing revealed significant improvement in scores between the second baseline and immediate post H-GRASP assessments with an effect size of 0.944 (Table 2). No significant changes were observed between initial and baseline assessments or between immediate post H-GRASP and the three and six month follow-up assessments (Table 4).

There was a significant main effect for upper limb use captured by the MAL (SM = 15.76, *p =* 0.003) and grip strength (SM = 18.47, *p =* 0.001) across the five time points. *Post hoc* Wilcoxon testing revealed significant improvement in scores between baseline and immediate post H-GRASP assessment for both the outcomes. The effect sizes for the MAL and grip strength were 0.789 and 0.947, respectively. No significant changes were observed between the initial and baseline assessments for either outcome. No significant changes were observed in the MAL or grip strength between the immediate post H-GRASP and the three month follow-up assessment. No significant changes were observed in the MAL between the post H-GRASP and the six month follow up assessment. However, a significant improvement in grip strength was observed between immediate post H-GRASP testing and the six month follow-up.

**Figure 3:**
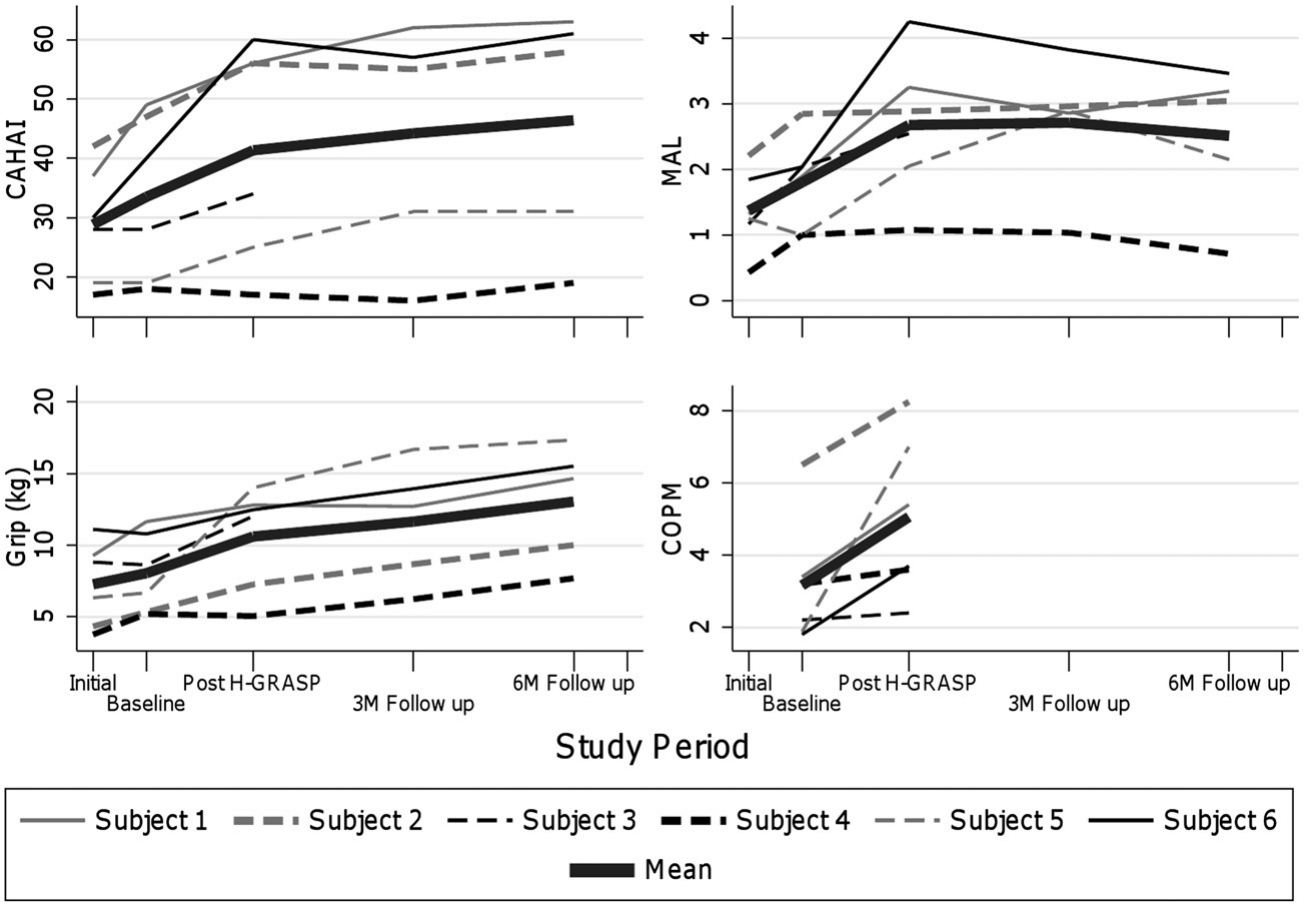
Participant outcome measure profiles. This graph displays clinical measure scores across the study period by participant. CAHAI: Chedoke Arm and Hand Inventory (0–63); MALQ: Motor Activity Log Quality of Movement Scale (0–5); Grip: Grip Strength; COPM: Canadian Occupational Performance Measure (1–10)

In addition, the Wilcoxon test revealed a significant improvement in COPM scores between the baseline and immediate post H-GRASP time points (*p =* 0.03) with an effect size of 0.789. These scores represented improvements in self-care, leisure and work activities. The activities identified as important to participants and their corresponding baseline and post H-GRASP ratings of performance are displayed in Table 5. Figure 3 displays the outcome measure profiles over the study period of the participants who completed the eight week H-GRASP. The participant with the lowest affected upper limb grip strength, function and use scores at initial testing also showed minimal change in any measures across the study periods (Participant 4).

**Table 5.** COPM activities and scores by participant.

Participant	Activities deemed important	Baseline COPM score	Post H-GRASP COPM score
1	Crocheting	3.4	5.4
	Knitting		
	Using dustpan		
	Cutting food		
	Driving		
2	Baking	6.5	8.3
	Cutting meat		
	Slicing food		
	Playing cards/scrabble		
3	Painting	2.2	2.4
	Dressing		
	Weeding		
	Household tasks		
	Using computer		
4	Feeding	3.2	3.6
	Dressing		
	Using tools		
	Writing		
	Driving lawnmower		
5	Using fork	1.9	7.0
	Using fishing rod		
	Buttoning		
	Picking up grandchild		
6	Styling hair	1.8	3.7
	Cooking		
	Volunteering		
	Returning to work		
	Writing		

## Discussion

This pilot study examined the feasibility of a novel upper limb exercise program for individuals living in the community. The feasibility thresholds for 6 out of 9 indicators were surpassed in our study and 9 out of 9 indicators were surpassed among participants who completed the eight week intervention. This suggests that H-GRASP may be a feasible program for individuals living at home and less than one year post stroke. Our findings also provide preliminary evidence that H-GRASP may promote improvements in affected upper limb function, use and strength.

The H-GRASP exercise component was feasible for the majority of participants who completed the eight week program as five out of six participants exceeded the minimum exercise targets by an average of 157 minutes per week. It should be noted that only 75% of individuals who enrolled in the study finished the eight week H-GRASP. Importantly, one individual dropped out of the program because he did not find the exercises sufficiently challenging, despite having moderate arm and hand impairments. It is possible that the exercises for this individual were initially set or progressed at too slow of a pace. This highlights the importance of providing each participant with a “just right challenge” which is consistent with the literature on maximizing brain plasticity.[30]

Following the completion of H-GRASP, we found statistically significant improvements in all our primary and secondary outcome measures. Specifically, we found group mean change scores of 7.8 points for the CAHAI, 0.9 points for the MAL, 2.6 kilos for grip strength and 1.9 points on the COPM. The recorded MCID values for these measures are 7.1 points (CAHAI),[22] 1.0 point (MAL),[31] 5.0 kilos (grip strength),[31] and 1.7 points (COPM).[32] The majority of our measures approached or surpassed the clinically meaningful thresholds and were then maintained. Effect sizes ranged from medium to large; however, the effect sizes should be interpreted with caution as they may be overinflated due to the lack of control group.[33]

The exercise component of the H-GRASP is grounded in evidence-based principles of intensity, repetition and task-oriented practice. The follow-up phone call content was heavily weighted on exercise monitoring and progression. The H-GRASP protocol was also unique however in that it included a variety of behavioural strategies to increase adherence to the program and encourage transfer of functional gains into use of the affected upper limb. The observed improvements in all of our clinical measures were maintained over the six month follow-up period. This suggests that individuals did translate functional gains into greater use of their upper limb. Interestingly, grip strength even continued to improve after the intervention had ended. The observed increase in grip strength in our study may further suggest that participants were continuing to incorporate their upper limbs into daily activities, and supports the known relationship between upper limb strength, function and upper limb arm use.[4,6] Three participants did improve greater than the MCID of 1.0 points on the MAL.[31] and two of these individuals increased their score to 3.0 on the MAL. A score of 3.0 on the MAL has been identified as clinically meaningful as it represents use of the affected limb without assistance from the other limb.[34] Interestingly, the individual who completed the eight-week program but did not adhere to the exercise target of 360 min/week approached or surpassed the MCID values on the CAHAI, MAL and COPM. A recent study noted that overall exercise session time may not be the most accurate measure of intensity.[35] Future research might consider the number of repetitions of active movement as a measure of intensity in conjunction with the errors made (an indication of the challenge). Alternatively, the intensity of exercise requested (i.e. 60 minutes per day 6 days/week) may be more than sufficient post stroke when there is a focus on task-specific, challenging repetition in combination with behavioural strategies to facilitate upper limb use. Also, only one participant who completed the 8 week intervention did not perform a task goal every week. Interestingly this individual had the greatest functional impairment and is the one participant who made little improvement. Extra care must be taken to help participants at lower levels of function set functionally appropriate goals that are achievable and therefore motivating.

Finally, responses in the COPM revealed that the wide range of activities participants perceived as important were improved as a result of H-GRASP (Table 5). Examples of improved activities noted by participants included feeding, styling hair, knitting and volunteering. This highlights the vast number of daily tasks that require the use of the upper limb and also emphasizes the uniqueness of upper limb use. Moreover, upper limb use may be responsive to behavioural strategies that incorporate client-centred upper limb goals due to the client-specific nature of upper limb use. Creating goals that are unique and meaningful to the individual may be an effective strategy for facilitating change in upper limb use.

Our intervention included a combination of behavioural strategies and thus we cannot tease out the impact of any one strategy. Literature suggests that behavioural change programs that include self-monitoring in combination with other self-regulation techniques such as goal setting, behavioural goal review and feedback are particularly effective for increasing health behaviours.[14]

Telerehabilitation for individuals post stroke is an emerging yet still understudied mode of service delivery. A recent Cochrane meta-analysis with three trials for the upper limb reported no adverse events and did not observe any differences between the tele-rehabilitation program and face to face rehabilitation which suggests that tele-rehabilitation may be a viable alternative mode of service delivery.[36] In our study, two participants experienced pain during the intervention period however the therapists were able to assist the participants to eventually complete the exercises without pain. In addition, exercise monitoring and progression by phone decreased the need for direct therapist time thereby increasing the resource-efficiency of H-GRASP. Weekly phone sessions on average took only 10–20 minutes. The current evidence regarding telerehabilitation for upper limb function combined with findings from our study suggest that programs able to monitor and progress participants by phone or internet may be suitable for rural settings where clients may have limited access to community services. Indeed, transportation concerns was the largest non-medical recruitment barrier in the EXCITE trial and is consistently cited as one of the most serious barriers to community participation after stroke.[37,38]

### Limitations

A major limitation of the study design is the small sample size and lack of an independent control group. However, this study included a one month baseline period over which participant scores were stable. Another limitation of the design is the increased potential for selection bias. It is likely that individuals who agreed to be in this study have greater motivation to exercise. Adherence to exercise targets of 60 minutes per day, six days a week may be lower in the general stroke population. Future studies examining exercise dose response using alternative measures of intensity may inform more specific exercise recommendations that require shorter time commitments. In addition, the use of person-centred behavioural techniques to maintain adherence and motivation to exercise may be even more pertinent for the general population. H-GRASP is suitable for individuals with mild to moderate impairment as the H-GRASP was designed to provide a specific and sufficient level of challenge to maximize brain plasticity. We obtained a recruitment rate of 26%. This is on par with the recruitment rate of 19% for the original GRASP study.[9] and larger than the recruitment rate of 6% for the EXCITE trial.[37] We only used self-report measures to capture adherence to exercise, changes in arm use and as a source of feedback to the participants during the H-GRASP intervention. Monitoring exercise behaviour using wearable devices may provide a more objective measure of exercise adherence and intensity. In addition, daily use with wearable devices might provide a more objective measure of arm use and increase the participants’ motivation to incorporate their upper limbs into daily activities. Finally, only two participants received assistance or support to perform the H-GRASP protocol from family and caregivers. Involvement of caregivers was associated with better upper limb functional outcomes in the original GRASP clinical trial.[9] Caregiver support is an important factor that warrants further examination in future studies exploring the efficacy of the H-GRASP protocol.

## Conclusions

In summary, the H-GRASP was feasible for our participants when they were sufficiently challenged by the exercise program. Our participants showed sustainable improvements in upper limb function, upper limb use, grip strength and occupational performance following the H-GRASP program. Our results suggest that a repetitive, task-oriented home exercise program that utilizes telephone supervision may be an effective method for the treatment of the upper limb following stroke. Care should be taken to ensure the exercise program is challenging for the participants. Moreover, an exercise program that utilizes behavioural change strategies may help overcome learned non-use among community dwelling individuals less than one year post stroke and thus promote transfer of exercise gains into sustainable use of the upper limb. Future studies that utilize a control group and objective measures of upper limb use are warranted to further examine the efficacy of the H-GRASP protocol.
